# Effect of curing distance for cure depth in composite resin

**DOI:** 10.6026/973206300191353

**Published:** 2023-12-31

**Authors:** Shantun Malhotra, Rubaldeep Kaur, Parneet Kaur Saroa, Kiranjot Kaur, Komaldeep Kaur Sandhu, Vani Thukral

**Affiliations:** 1 SGRD Dental College and Hospital, Amritsar, Punjab, India

**Keywords:** Light curing units, composites, distance, curing depth

## Abstract

The effect of altering the distance between light tip and outer layer of composite (DLR) on the depth of composite cure with a range of low to high light intensity curing lamps and with different types of light unit is of interest. Three LED units (T= Freelight,U = Ultrablue IS, V = Coltolux LED, one PAC unit (S=PAC) and three halogen light-curing units (P = XL2500, Q = HelioluxVL, R = Visiolux) were analyzed. A human molar tooth that was separated mesio-distally to buccal and lingual halves was used to create a natural tooth sample. Data shows that the depth of cure for the composite material decreased as the distance between the repair and the light source increased. There was no appreciable difference in performance between the LEDs and the other kinds of curing lamps as the distance between them grew.

## Background:

More compact, longer-lasting restorations are the aim of adhesive based operative dentistry [1, 2]. The time since the light-cured composites were first introduced to consumers over three decades ago, innovations in adhesion along with polymerization, development of novel materials, and application of sensible or "restricted intervention" restoration procedures have completely changed the field of dentistry [3- 4]. One component of an appropriate clinical polymerization procedure is the light for curing. The result is greatly influenced by the skills of the dentist, the preparation of the composite substance (form and loading of filler, composition of resin, initiator system ,shade and opacity, etc.), the adhesive arrangement, and the method used for polymerization technique [4,5]. The objectives of dental photo curing include attempting to minimize the consequences of tissue, material, tooth heating up and transformation shrinkage stress while achieving consistent maximum conversion to complete depth in the shortest feasible radiation time [5 6,7,8].

Dental restorations are now more successful because to the use of light-cured resin composites [1- 3]. A number of variables, such as power density, exposure duration, resin color, filler size, and filler loading, affect how well composite materials cure. Some authors propose an inverse square law, according to which light intensity falls according to the square of the distance between the light source and the outer layer of the beneficial material (DLR) [9-10,11, 12]. The curing duration, dimensions, the spot, and positioning of the light tip, the depth, shading and chemical structure of the substance that is being cured, as well as the wavelength and strength of light output emanating from the curing unit all affect the degree of polymerization [13,14]. A decrease in the strength of bonds and a rise in water intake and dissolution are possible outcomes of partial polymerization. Numerous factors, including changes in the mains voltage, timer preciseness, and degradation in the filter, bulb, reflector, fiber-optic lead, and tip, can affect the amount of light that a halogen curing unit produces [15-16, 17,18].

A survey conducted in the United Kingdom (UK) found that sixty-three percent of curing lights generated low light output, slightly more than the 45.5 percent of lights that did not meet specifications found in an American survey [19- 20,21]. This is regardless of the prerequisite for sufficient cure. The DLR and separating medium (typically air alone, or a combination with light tip envelope or auxiliary plastic matrix) both affect how much light is able to illuminate the material that has to be cured [22- 23]. Light intensity decreases with increasing DLR and some authors refer to an inverse square law relationship [24-25].

Nowadays, there are different types of light curing units available for dental professionals. These include different LED light curing units, halogen light curing units, plasma arc curing unit (PAC) [20-24]. The light output in these light curing units is different. Therefore the positioning and distance of light tip affect the curing process and depth of curing [19-23]. Dentists should also have knowledge including about the effect of curing distance on depth of cure of composite resin using different light curing units of different intensities. They would be better able to choose the proper thickness of a composite layer that can be consistently treated in a clinical environment if they knew how much of a specific shade of light-activated composite material could be properly cured. Additionally, this information would provide important benchmark data on the precise cure depth of several light-initiated composite materials that dentists frequently use [4-11]. Therefore, it is of interest to determine the effect of altering the DLR on the depth of composite cure with a range of low to high light intensity curing lamps and with different types of light unit. By adjusting the DLR, the experiment aimed to examine the effects of different light cure units and light intensities on the curing depth of composites.

## Materials and Method:

Following light curing units were selected to provide lights of low to high output. Rather than being used to evaluate newly constructed units, these units were chosen to reflect the variety of LCUs currently in application in clinical settings. Additionally, a wider variety of light outputs could be achieved with this method (Table 1).

Three LED units:

T= Free light (3M/ESPE)

U= Ultra blue IS (DMC),

V= Coltolux LED (ColteneWhaledent)

One PAC unit:

S = PAC

Three halogen light-curing units:

P=XL2500 (3M/ESPE),

Q= HelioluxVL,

R= Visiolux, 3M

The intensity of light from LCUs of lesser power (P-R), was measured with a Demetron 100 radiometer (Demetron Research Corp., USA), and the intensity of light from LCUs of higher power (S-U), was measured with an SDI radiometer. By putting the tip of light probe over the radiometer's light cell until the maximum light intensity value appeared on the metre, light intensity was measured. As advised by Hansen and Asmussen (1993), every measurement was collected inside five seconds of activation. The intensity of visible light (VL) was obtained for each LCU at 0 mm, 1 mm, 2 mm, 3 mm, 4 mm, 5 mm, 7 mm and 10 mm.

A human molar tooth that had been separated mesio-distally to buccal as well as lingual halves was used to create a natural tooth framework, which had been proven to be useful in an earlier study [7,9]. The pulp chamber was included in an 8 mm deep cavity that was created apically from the occlusal tooth surface. Since the tooth utilised in this investigation was acquired from market established prior to the implementation of the current consent specifications, ethical authorization was not necessary. To create a smooth surface and allow the light-cure tip to approximate the restoration surface closely, the cusps of teeth have been reduced onto the occlusal surface. The occlusal surface had a cavity that measured about 4 mm in diameter and 8 mm in depth when it was assembled again.

The tooth was maintained moist in between experiments, and no separator was needed in the prepared cavity to aid in dismantling. The adjustable table of the experiment set up held the natural tooth framework in place. The table was fastened to the fixed component of experiment set up, into which the light-cure unit's tip was secured with clamps.

The DLR could be modified in increments of 1 mm from 0 to 15 mm by rotating the screw. A radiometer was used to obtain five-meter readings for every light-cure unit's intensity prior to the depth-of-cure accurate measurements. There was assessment of light intensity

Every unit was then utilised for curing 70 composite posterior restorations. The tip of light source was placed in contact with the natural tooth, having minimum gap with composite surface to ensure no matrix was needed. Throughout the investigation, a single shade (A2 dentine) of a widely accessible, well-liked composite resin material for restoration (Herculite XRV, Kerr) was utilised, as it has been shown that depth of cure is significantly influenced by the color and transparency of the material.

Since oxygen resistance of the composite resin's surface was thought to be negligible and would be an unaltered for all specimens, it was not taken into consideration. Before the placement, the composite resin was stored at room temperature because it has been demonstrated that the polymerization of composite resin through different LCUs is influenced by temperature at the time of insertion.

35 restorations were cured for 20 seconds and an additional 35 restorations were cured for 60 seconds using the three halogen light units (lights P, Q, and R). Further five readings of light output were then obtained. Initially the readings were obtained when the light tip was in contact with sensor of radiometer. Then finally, readings were obtained when light tip was in contact with natural tooth framework. The PAC light (S) was used to cure restorations for three seconds. Owing to their higher outputs at a 20-second cure time, the LED light curing units (lights T, U, and V) were evaluated at 20 seconds duration of curing time for all 70 specimens.

The LCUs of lower intensity (P,Q,R) were used for curing of composite restorations at distances of 0mm,1mm,2mm,3mm,4mm,5mm and 10mm while LCUs of higher higher intensity (T,U,V) were used for curing at distances of 5mm,10mm and 15 mm. Following each restoration's light curing period, each model had been dismantled and one operator used a scraping technique in accordance with ISO 4049.18 to determine the composite's depth of cure. This required removing anything that a flat plastic device could remove. A digital vernier calliper (Digimatic, Mitutoyo, Japan) was used to measure the least separation the base of the remaining cured and surface of composite. Numerous groups have made extensive use of this technique.

## Statistical analysis:

ANOVA and the linear correlation coefficient were used to analyse the data and compare the relationship between separation distance and depth of cure.

## Results:

The VL intensity readings from the curing lights at various DLR levels, as determined by a radiometer, are shown in (Table 2).

The thickness of the completely cured composite is measured using the ISO 4049 test. Table 3 displays the calculated cure depths for every spacing and light source. When the visible spectrum light source's power was at its lowest, the depth of cure was at its lowest as well. The most deeply cured restoration was produced by the VL light at the maximum intensity. The degree of cure was lessened with higher DLR values. There was more substantial polymerization after 60 seconds as opposed to 20 seconds. For all three halogen lamps, the average rise in cure depth between 20 and 60 seconds was 1.410.07 mm, regardless of the lights' spatial spacing. "The use of three LED light curing units produced comparable outcomes to halogen lamps and PAC (Table 2), where the depth of cure decreased as the DLR increased.

Before and after every experiment, the visible light (VL) output was monitored to make sure that the light intensity remained constant throughout the trials. Five measurements were taken before each experiment began and five measurements were taken after it was over. The average of these measurements was then determined. The following data shows oscillations in the mean levels of light emission before and after each experimental effort. Light P has an intensity of 427-431 milli watts per square cm. Light Q has an intensity of 188-186 milli watts per square cm. Light R has an intensity of 80-84 milli watts per square cm. Light S has a milli watt-per-square-centimeter intensity of 11-61-1170. Light T has intensity of has an intensity of 1920-1930. Light U has intensity of 845 to 880 milli watts per square cm. Light U has intensity of 850 to 890 milli watts per square cm. Throughout the trial, there were no discernible variations in the mean light output levels for any light source (; ANOVA, P>0.05). These results show that each light unit's performance remained constant over the course of the investigation, with lights B and C showing somewhat less clinical efficacy than the threshold

## Discussion:

This study was therefore conducted to determine the effect of altering the DLR on the depth of composite cure with a range of low to high light intensity curing lamps and with different types of light unit. By adjusting the DLR, the experiment aimed to examine the effects of different light cure units and light intensities on the curing depth of composites. Seven distinct light-curing devices that are already in use in clinical settings were used in the depth-of-cure trial.

In this study when the visible spectrum light source's power was at its lowest, the depth of cure was at its lowest as well. The most deeply cured restoration was produced by the VL light at the maximum intensity. The degree of cure was lessened with higher DLR values. There was more substantial polymerization after 60 seconds as opposed to 20 seconds. For all three halogen lamps, the average rise in cure depth between 20 and 60 seconds was 1.410.07 mm, regardless of the lights' spatial spacing. "The use of three LED light curing units produced comparable outcomes to halogen lamps and PAC, where the depth of cure decreased as the DLR increased.

These results are consistent with a study [11], which found that the main parameters influencing the polymerization process are not energy thickness but rather the composition and intrinsic qualities of the composite resins. However, the fixing value depths of Range dental composite are substantially shallower. Radiation exposure duration, light intensity, filler and sap technology, and other factors all affect the therapeutic depth of light-activated tar-based composites. This study's trials showed a relationship between a reduction in the light's impact and the depth of the fix and the distance between the light source and the supporting material's outer layer (DLR) [12]. The decline was correlated with the distance. Prior research has demonstrated a relationship between the depth of cure and the logarithm (base 10) of the average light intensity as the DLR rises. Our analysis for VL output provides additional confirmation of our association [13]. It was shown in a study [11] that as the distance rose, the depth of cure decreased progressively and linearly. The notion that the light intensity did not follow the inverse square rule within the parameters of this investigation (0.1-1 mm) was supported by another study [15]. DLRs with a range of 0 to 15 mm were found in a study [16] to have no effect on the surface hardness of composite resin. A study, however, revealed that DLRs larger than 20 mm dramatically reduced the depth of cure.

The investigation found that using light with a modest output (unit R in this experiment, 80 mW/cm2), a composite resin with a thickness of 1.8 mm may be solidified in about 20 seconds without detaching from the composite material. At this point, only about half of its thickness may be said to be entirely healed with confidence. This light source was only able to cure around 0.7 mm of composite material (1.4 mm according to the ISO 4049 test) at a spacing of 10 mm. By doubling the curing time from 20 to 60 seconds, the average depth of cure rose by about 1.4 times. This is consistent with earlier data where there is no space between the tip of the light source and the composite material [12-15].

Since all that is needed to determine the depth of cure is a scraping tool, a dental clinic may carry out the procedure. By exposing them to light, dentists may use the ISO method to precisely determine how long different composite materials will take to cure. "Once a baseline value has been established, the dentist often uses this procedure to evaluate the degree of curing, assuring the best possible performance from the resin-based composite and the curing light. The intensity of curing light can be measured using a commercial light metre, but not the other way around. Because resin-based composites come in such a wide range of compositions, colours, and translucencies, curing light intensity alone is not enough to achieve a complete cure [9,13].

Dentists may use the information gathered from the ISO technique to accurately calculate how long a particular resin-based composite and light source needs to cure. A specimen is deemed cured, in accordance with ISO standards, once uncured composite material have been removed using a plastic spatula, removing half of its length. Some research have shown a considerable decrease in the hardness of the cured composite from the top to the lower section of the sample, whereas many studies use the residual length after removing uncured material as a measure of the amount of curing. Since the residual length was used to gauge the depth of cure, inadequate polymerization might have a negative impact on clinical performance. The ISO has established a more stringent standard by defining the depth of cure as half of the remaining length in order to emphasize prudence. After evaluating the depth of cure, a suggestion was made to ascertain the proportion of the scraped specimen that remained [9].

In this study the intensity of visible light (VL) from different LCU as recorded by radiometer at different distances are shown in Table 1. It was observed that there was decrease in intensity of VL from each LCU as the distance increased between LCU and radiometer. The change was statistically significant in each LCU. The analysis of variance (ANOVA) revealed a statistically significant association between DLR and intensity. The variation in distance between the two LED lights (either 0 or 5 mm) had no noticeable impact.

To ascertain the degree of cure, a study [13] values were compared to the outcomes of hardness tests, translucency changes, and double-bond conversion. A data analysis [10] revealed that employing 50% of the scraped length produced depth-of-cure estimations that were either more conservative or equivalent to those determined by hardness or the degree of double-bond conversion using infrared spectroscopy. Consequently, by using the ISO approach, the majority of resin-based composites should go through enough polymerization.

The quantity of light that can penetrate a material's interior layers decreases with further exploration, which limits the amount of polymerization that may occur. The fillers' dimensions determine how much of the material cures. Fillers in resin-based composite materials cause the phenomena of light scattering. Because of the scattering effect, the composite's light transmission decreases as the fillers' particle sizes become closer to that of the activating light [14].

Not a single curing lamp's VL output changed noticeably during the course of the testing period. It is not possible to explain the observed differences in cure depth with increasing DLR to variations in output intensity brought on by changes in mains voltage, deterioration of bulbs, reflectors, fibre optics, or state of the light source tips. Consistent with a study, inquiries [15-17,18], there was a comparable decrease in the depth and intensity of cure of visible light (VL) as the DLR (dose-to-light ratio) increased.

## Conclusion:

Data shows that the depth of cure for the composite material decreased as the distance between the repair and the light source increased. There was no appreciable difference in performance between the LEDs and the other kinds of curing lamps as the distance between them grew.

## Figures and Tables

**Table 1:** Distribution of total study specimens (490) in seven categories

**S. No**	**Category**	**LED unit**	**No of specimens**
1	P	XL2500 (3M/ESPE),	70
2	Q	Helio luxVL	70
3	R	Visiolux,3M	70
4	S	PAC	70
5	T	Free light (3M/ESPE)	70
6	U	Ultra blue IS (DMC)	70
7	V	Coltolux LED (Coltene Whaledent)	70

**Table 2:** Data showing mean visible light radiometer readings (mW/cm2) for the halogen (P-R), PAC (S) and LED curing lights (T-V) at the different separation distances between radiometer and light tip (n=10)

	**Visible light radiometer readings (mW/cm2)**						
**Separation in mm**	**Unit P**	**Unit Q**	**Unit R**	**Unit S**	**Unit T**	**Unit U**	**Unit V**
0	531	207	93	1319	2031	863	872
1	487	205	83	1249	1957	804	813
2	447	201	79	1118	1817	759	768
3	395	187	63	1077	1476	708	717
4	221	175	53	985	1188	613	621
5	195	165	41	895	1032	533	547
7	101	134	34	785	987	448	453
10	87	98	27	703	913	270	261
	<0.001	<0.001	<0.001	<0.001	<0.001	<0.001	<0.001

**Table 3:** Data regarding depth of polymerization on placing tip of LCU in contact with composite at different distance between light tip and composite specimen

	**Polymerization depth in mm**									
**Distance of separation mm**	**20s unit P**	**20s unit Q**	**20s unit R**	**60s unit P**	**60s unit Q**	**60s unit R**	**3s unit S**	**20s unit T**	**20s unit U**	**20s unit V**
0	3.29	2.91	1.92	4.31	3.91	2.4	4.51	5.22	5.24	5.25
1	2.91	2.77	1.89	3.93	3.93	2.35	4.5	-	-	-
2	2.62	2.59	1.68	3.7	3.88	2.36	4.25	-	-	-
3	2.63	2.54	1.64	3.57	3.75	2.34	4.15	-	-	-
4	2.56	2.52	1.62	3.63	3.62	2.17	3.83	-	-	-
5	2.52	2.46	1.58	3.56	3.56	2.14	3.64	5.33	5.02	5.02
10	2.31	2.34	1.45	3.45	3.42	2.1	3.72	4.27	4.25	4.25
15	-	-	-	-	-	-	-	3.17	3.62	3.63
	<0.01	<0.01	<0.01	<0.01	<0.01	<0.01	<0.01	<0.01	<0.01	<0.001
